# Phthalate Metabolites and Their Relationship with Abdominal and General Obesity: Evidence from the Aragon Workers’ Health Study (AWHS)

**DOI:** 10.3390/nu17111869

**Published:** 2025-05-30

**Authors:** Jordan Akritidis, Diana María Mérida, Carolina Torrijo-Belanche, Belén Moreno-Franco, Sofía Gimeno-Ruiz, Jimena Rey-García, María Morales-Suarez-Varela, Pilar Guallar-Castillón

**Affiliations:** 1 Department of Preventive Medicine and Public Health, School of Medicine, Universidad Autónoma de Madrid, Arzobispo Morcillo 4, 28029 Madrid, Spaindianamerida26@gmail.com (D.M.M.); 2 CIBERESP (CIBER of Epidemiology and Public Health), 28029 Madrid, Spain; maria.m.morales@uv.es; 3 Fundación Teófilo Hernando, Las Rozas de Madrid, 28290 Madrid, Spain; 4 Department of Preventive Medicine and Public Health, Universidad de Zaragoza, 50009 Zaragoza, Spain; carolinatorrijob@gmail.com (C.T.-B.); belenmorenofranco@gmail.com (B.M.-F.); 5 Instituto de Investigación Sanitaria Aragón, Hospital Universitario Miguel Servet, 50009 Zaragoza, Spain; 6 CIBERCV (CIBER de Enfermedades Cardiovasculares), 28029 Madrid, Spain; 7 Veterinary School, Universidad de Zaragoza, 50013 Zaragoza, Spain; gimenoruizsofia@gmail.com; 8 Department of Internal Medicine, Hospital Universitario Rey Juan Carlos, Instituto de Investigación Sanitaria-Fundación Jiménez Díaz (IIS-FJD), 28933 Móstoles, Spain; jimena.reygarcia@gmail.com; 9 Departament de Medicina Preventiva i Salut Pública, Ciències d’Alimentació, Toxicologia i Medicina Legal, Universitat de València, 46003 Valencia, Spain; 10 Instituto Madrileño de Estudios Avanzados en Alimentación (IMDEA)-Food Institute, Campus de Excelencia Internacional Universidad Autónoma de Madrid + Consejo Superior de Investigaciones Científicas (CEI UAM + CSIC), Carretera de Cantoblanco 8, 28049 Madrid, Spain

**Keywords:** phthalates, abdominal obesity, general obesity, DEHP, environmental epidemiology, nutritional epidemiology

## Abstract

Background/Objectives: Phthalates are endocrine-disrupting chemicals that are commonly used in plastic consumer products and food packaging, with growing evidence suggesting that they have a potential role in obesity. This study aimed to investigate the association between urinary concentrations of phthalate metabolites and both general and abdominal obesity among adult males in Spain. Methods: We analysed data from 1124 male participants of the Aragon Workers’ Health Study (AWHS) collected between 2011 and 2014 in Zaragoza, Spain. Eleven urinary phthalate metabolites were measured and adjusted for creatinine levels. Multivariate logistic regression models were used to evaluate associations between phthalate exposure and general and abdominal obesity, controlling for dietary and lifestyle factors. Dose–response relationships were explored using restricted cubic spline models. Results: Higher urinary concentrations of di(2-ethylhexyl) phthalate (∑DEHP) and two of its metabolites—mono-(2-ethyl-5-oxohexyl) phthalate (MEOHP) and mono-(2-ethyl-5-hydroxyhexyl) phthalate (MEHHP)—were significantly associated with general obesity. The adjusted odds ratios were: ∑DEHP [OR = 1.26; 95% CI: 1.01, 1.58], MEOHP [OR = 1.24; 95% CI: 1.00, 1.53], and MEHHP [OR = 1.26; 95% CI: 1.03, 1.55]. In contrast, mono-isobutyl phthalate (MiBP) was inversely associated with abdominal obesity [OR = 0.73; 95% CI: 0.57, 0.93]. Conclusions: These findings suggest a positive association between exposure to DEHP and its metabolites and general obesity. This highlights the potential importance of environmental exposures as modifiable factors in obesity prevention and supports the need for further investigation in nutritional and public health contexts.

## 1. Introduction

Obesity is a multifactorial condition associated with an elevated risk of many chronic diseases and all-cause mortality [1]. While excessive caloric intake and insufficient physical activity are key risk factors for obesity, environmental exposure to endocrine disruptors such as phthalates may also play an important role. Phthalates are organic lipophilic compounds used mainly as additives to soften plastics in food packaging [2], vinyl flooring [3], medical equipment [4], water bottles [5], and personal care products [6]. Since they do not covalently bind to plastic, phthalates easily leak into surrounding air, water, and food resulting in inhalation, ingestion, and dermal absorption [7]. Subsequently, they are quickly metabolised and excreted via urine and faeces.

Phthalate exposure is ubiquitous and industrial use is extensive with between 6 and 8 million tons of phthalates being manufactured globally each year [8]. This exposure has been linked to type 2 diabetes [9], metabolic syndrome [10], thyroid function [11], low birthweight [12], attention deficit hyperactivity disorder [13], endometriosis [14], decreased testosterone [15], decreased anogenital distance [16], and hearing disorders [17]. Infants and children in particular appear to be most susceptible to the endocrine-disrupting properties of phthalates especially during early growth [18,19]. Consequently, attempts have been made to limit the use of several phthalates in children’s toys in many countries including China, the world’s largest producer and consumer of phthalate-containing products [20,21].

Phthalate exposure may play a key role in the development of obesity, although its association differs significantly between males and females, as well as between childhood and adulthood [22,23]. The National Health and Nutrition Examination Survey (NHANES) conducted in the United States provides key data on the association between phthalate exposure and obesity in the general population [22,24,25,26]. While among children, studies show that prenatal phthalate exposure is positively associated with higher body mass index (BMI) and waist circumference (WC) [27,28,29], the effects of chronic exposure among adults is less clear due to a lack of longitudinal studies, high heterogeneity between studies, and inconsistent results. For instance, specifically among men, several NHANES studies found that certain phthalates of high molecular weight were significantly associated with either abdominal or general obesity [24,25,30], while other large studies reported non-significant or inverse associations between phthalates and body weight outcomes in men [23,31].

Epidemiological studies including NHANES have reported positive associations between di(2-ethylhexyl) phthalate (DEHP) metabolites and obesity in men [22,24,25]. Among women, DEHP has also demonstrated positive associations with obesity and body weight outcomes in several studies [31,32,33]. Exposure to DEHP is high in the general population as it is found in high quantities in plastic food packaging [20] and personal care products [34]. Phthalates including DEHP likely act by interfering with energy metabolism and adipose tissue structure [35], which in turn contributes to the development of obesity.

Given the widespread exposure of the general population to phthalates and the conflicting evidence in the current scientific literature, further research into the obesogenic effects of phthalates in adults is clearly warranted. We hypothesise that phthalates are positively associated with both abdominal and general obesity. This study aims to investigate the association between urinary phthalate metabolite concentrations and abdominal and general obesity in adult Spanish males.

## 2. Materials and Methods

### 2.1. Study Design and Participants

This is a cross-sectional study using data from the Aragon Workers’ Health Study (AWHS), whose design and methodology has been detailed elsewhere [36]. The AWHS is a prospective cohort study, based on data from the annual physical examinations of 5678 workers in a large car assembly plant in Figueruelas, Zaragoza, Spain, aimed to investigate the determinants of the development and progression of subclinical atherosclerosis. From 2011 to 2014, participants aged 39–59 years (95.0% men) underwent subclinical atherosclerosis imaging and an interview with questionnaires on diet, behaviour and lifestyle factors. The current study utilises data from 2133 participants with blood and urine samples. We excluded females (*n* = 114), those with a personal history of cardiovascular disease (*n* = 27), those without urinary creatinine measurements (*n* = 837), those with extremely diluted or concentrated urinary creatinine (less than 30 mg/dL or more than 300 mg/dL) (*n* = 30), and those with a BMI of <18.5 kg/m^2^ (*n* = 1). The final analyses included 1124 participants. We report these findings in line with the Strengthening the Reporting of Observational Studies in Epidemiology (STROBE) guidelines [37].

### 2.2. Endpoints

There were two endpoints in this study. (1) Abdominal obesity, which in males is defined as having a WC of >102 cm (42 inches) [38]; and (2) general obesity, defined as having a BMI of ≥30.0 kg/m^2^ [39]. Anthropometric data (WC, weight, and height) were measured by trained physicians and nurses using standardised procedures. A total of 40 participants had missing values for WC but had recorded BMI.

### 2.3. Urinary Phthalate Metabolite Measurements

Phthalate metabolites were quantified in urine using high-performance liquid chromatography in tandem with mass spectrometry (LC-MS/MS) and expressed in ng/mL. Valid urinary concentrations were obtained for 11 phthalate metabolites: monoethyl phthalate (MEP); mono-isobutyl phthalate (MiBP); mono-n-butyl phthalate (MnBP); monobenzyl phthalate (MBzP); mono-(2-ethyl-5-oxohexyl) phthalate (MEOHP); mono-(2-ethyl-5-carboxypentyl) phthalate (MECPP); mono-(2-ethyl-5-hydroxyhexyl) phthalate (MEHHP); mono(2-carboxymethylhexyl) phthalate (MCMHP); mono-carboxy isononyl phthalate (cx-MiDP); mono-hydroxy-isodecyl phthalate (OH-MiDP); and mono-hydroxy-isononyl phthalate (OH-MiNP). The limit of detection (LOD) was established at 0.5 ng/mL for MEP, MBzP, MEOHP, MECPP, MEHHP, and MCMHP, and 1.0 ng/mL for MiBP, MnBP, cx-MiDP, OH-MiDP, and OH-MiNP. Nine of the eleven phthalate metabolites were detected in all 1124 participants, while cx-MiDP and OH-MiDP were detected in 981 (87.3%) and 977 (86.9%) participants, respectively. Values below the LOD were estimated by dividing the LOD by the square root of two [40]. In line with previous research [12,41], we excluded three phthalate metabolites with more than 75% of measurements below the LOD: mono-cyclohexyl phthalate (MCHP), mono-n-pentyl phthalate (MnPP), and mono-n-octyl phthalate (MnOP). The molar sum of DEHP metabolites (∑DEHP) was calculated from the sum of each metabolite divided by its molecular weight: MEOHP/292.33 + MECPP/308.33 + MEHHP/294.34 + MCMHP/308.33.

### 2.4. Creatinine Correction

We corrected urinary phthalate metabolite (in μg/g creatinine) and ∑DEHP (in μmol/g creatinine) concentrations for creatinine to account for urine dilution. The sample median was 151.25 mg/dL. We converted phthalate metabolite values from ng/mL to µg/dL by multiplying by 0.1, and creatinine values from mg/dL to g/dL by multiplying by 0.001. As a result, to obtain phthalate metabolite values, we divided each phthalate metabolite by creatinine and then multiplied each concentration by 100. To obtain DEHP values, we divided ∑DEHP by creatinine concentration and multiplied by 100.

### 2.5. Data Collection and Covariates

Demographic data and smoking status were collected via questionnaires. A 136-item semi-quantitative food frequency questionnaire (FFQ) [42] validated in Spain assessed dietary intake over the previous year to obtain alcohol and energy intake. Physical activity data were collected via the validated Spanish-language version of the physical activity questionnaire from the Nurses’ Health Study and Health Professionals’ Follow-up Study [43]. Hypertension was defined by having systolic blood pressure ≥ 140 mmHg or diastolic blood pressure ≥ 90 mmHg, or by taking hypertension medication [44]. Dyslipidaemia was defined by having total blood cholesterol ≥ 240 mg/dL, triglycerides ≥ 150 mg/dL, LDL ≥ 160 or HDL < 40 mg/dL, or by taking dyslipidaemia medication [45]. Diabetes was defined by having blood glucose ≥ 126 mg/dL, or by taking diabetes medication [44]. Participants were classified according to work type, as sedentary work (office) or manual labour (assembly line). Participants worked one of four shift schedules. There were two rotating shifts: morning–evening (06:00–14:00 and 14:00–22:00), and morning–evening–night (06:00–14:00, 14:00–22:00, and 22:00–06:00). There were two fixed shifts: central (08:00–16:00), and night (22:00–06:00). Workers on rotating shifts changed on a weekly basis.

### 2.6. Statistical Analysis

All eleven phthalate metabolites and ∑DEHP were categorised into quartiles and analysed by comparing successive quartiles to the first quartile using multivariate logistic regression. To calculate *p*-test for linear trend, quartiles were considered as a continuous variable. Correlations between each phthalate metabolite concentration were calculated using Spearman’s rank correlation test. Phthalate metabolites were also analysed as continuous variables after ln-transformation to improve normality. To assess dose–response between the metabolites and abdominal and general obesity, we depicted restricted cubic spline models (RCS). In alternative (sensitivity) analyses to correct for urinary dilution, we repeated the logistic models with ln-phthalate metabolites as dependent variables and adjusted for urinary creatinine. Data were analysed using STATA v18 [46] and *p*-values of <0.05 were considered significant.

### 2.7. Ethics and Consent

This study was approved by the Clinical Research Ethics Committee of Aragon (CEICA) (PI07/09). Informed consent was granted by participants in the AWHS who allowed the use of their data collected via the annual health exam, additional questionnaires on cardiovascular and lifestyle risk factors, and blood and urine samples collected for the study biobank. All participants belong to the AWHS cohort; therefore, prior to their inclusion, they signed a written consent form.

## 3. Results

### 3.1. Participant Characteristics

All participants were males of Spanish origin with an average age of 50.7 years (±3.7 years). Alcohol intake (g/day) was significantly higher among those with greater urinary concentrations of MEHHP and ∑DEHP, while energy intake (kcal/day) was significantly higher among those with greater urinary concentrations of MEOHP, MEHHP, and ∑DEHP. Most participants were manual labourers (87.5%) and worked the rotating morning–afternoon shift (61.3%). Greater urinary concentrations of MEOHP, MEHHP, and ∑DEHP were observed for manual workers in morning–afternoon shifts (Table 1).

### 3.2. Urinary Phthalate Metabolites

MEP had the highest median urinary concentration in our sample (100.9 μg/g creatinine). We also detected MiBP (16.3 μg/g creatinine), MnBP (13.5 μg/g creatinine), MBzP (5.5 μg/g creatinine), MEOHP (6.38 μg/g creatinine), MECPP (13.0 μg/g creatinine), MEHHP (12.2 μg/g creatinine), MCMHP (2.79 μg/g creatinine), OH-MiNP (8.16 μg/g creatinine), cx-MiDP (1.30 μg/g creatinine), and OH-MiDP (1.60 μg/g creatinine). Correlations between the four metabolites of DEHP (MEOHP, MECPP, MEHHP, and MCMHP) were high (Rho > 0.80), as was the correlation between cx-MiDP and OH-MiDP (Rho = 0.83). All other phthalate metabolites were weakly or non-correlated (Table 2).

### 3.3. Phthalate Metabolites and Abdominal Obesity

In total, 29.2% (317/1084) had abdominal obesity. In multivariate logistic regression analysis, MiBP showed an inverse and significant association with abdominal obesity when comparing extreme quartiles. We obtained consistent results when MiBP was considered as a continuous variable.

MEOHP was significantly higher in the second [OR = 1.99 (95% CI: 1.33–2.96)] and fourth [OR = 1.53 (95% CI: 1.02–2.30)] quartiles compared with the first quartile adjusted for covariates (*p*-test for linear trend = 0.249). We obtained consistent results when MEOHP was considered as a continuous variable.

MBzP was significantly higher in the second quartile compared with the first quartile adjusted for covariates, but this association did not hold for the fourth quartile and the *p*-test for linear trend was not significant.

**Figure 1:**
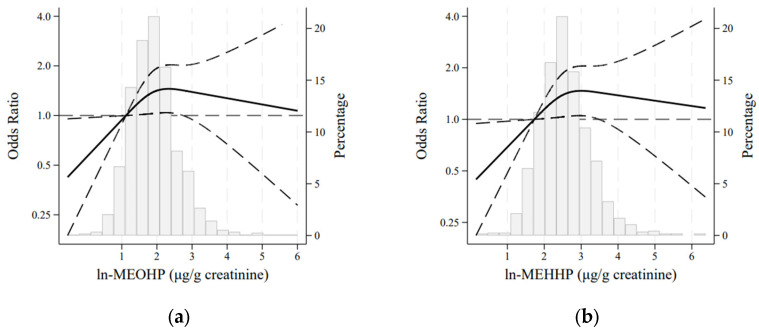
Adjusted restricted cubic spline models for the association between abdominal obesity and (**a**) ln-MEOHP and (**b**) ln-MEHHP in the AWHS cohort (N = 1084). Models adjusted for age, alcohol intake, physical activity, energy intake, smoking status, hypertension, dyslipidaemia, diabetes, work type, and work shift. The solid and long-dashed lines represent the estimated odds ratios and their 95% confidence intervals, respectively.

When the dose–response relationship was depicted using RCS, a positive but non-linear association was observed between ln-MEOHP and ln-MEHHP with abdominal obesity (Figure 1).

∑DEHP was marginally associated with abdominal obesity. The association was approximately 50% higher when the fourth quartile was compared to the first one. When considered as a continuous variable, the association was 26% higher for each unit increase in ln-∑DEHP (Table 3). In sensitivity analyses, similar but more conservative results were obtained (Table S1).

### 3.4. Phthalate Metabolites and General Obesity

In total, 21.9% (246/1124) had general obesity. In multivariate logistic regression analysis, MEOHP was significantly higher in the second [OR = 1.79 (95% CI: 1.15–2.76)] and fourth [OR = 1.70 (95% CI: 1.09–2.64)] quartiles compared with the first quartile and the *p*-test for linear trend was marginally significant (*p*-test for linear trend = 0.088). Additionally, we obtained an OR of 1.24 (95% CI: 1.00, 1.53; *p* = 0.047) for each unit increase in ln-MEOHP.

For MECPP, we obtained marginally statistically significant results when studied in quartiles [OR = 1.53 (95% CI: 0.98–2.32)] comparing the fourth with the first quartile (*p*-test for linear trend = 0.112). When considered as a continuous variable [OR = 1.23 (95% CI: 0.99–1.53; *p* = 0.062)] was obtained for each unit increase in ln-MECPP.

MEHHP was positively associated with general obesity, with an OR of 1.63 (95% CI: 1.06–2.52) when comparing the fourth quartile to the first quartile. This association was monotonic (*p*-test for linear trend = 0.041). Additionally, an OR of 1.26 (95% CI: 1.03–1.55; *p* = 0.027) was obtained for each unit increase in ln-MEHHP.

∑DEHP was marginally associated with abdominal obesity when studied in quartiles [OR = 1.54 (95% CI: 0.99–2.37)] when comparing the fourth with the first quartile (*p*-test for linear trend = 0.075). Similar results were obtained when considered as a continuous variable; an OR of 1.26 (95% CI: 1.01, 1.58; *p* = 0.038) was observed for each unit increase in ln-∑DEHP.

The remaining seven phthalate metabolites did not show significant associations with general obesity in these analyses (Table 4). In sensitivity analyses, similar results were obtained however MECPP reached statistical significance [OR = 1.63 (95% CI: 1.00–2.66)] when comparing the fourth with the first quartile (*p*-test for linear trend = 0.101) (Table S2).

**Figure 2:**
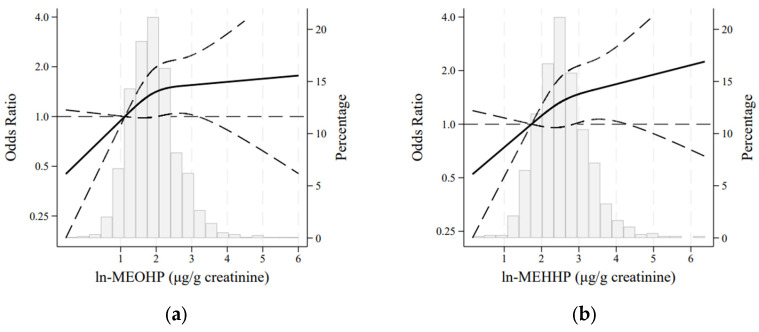
Adjusted restricted cubic spline models for the association between general obesity and (**a**) ln-MEOHP and (**b**) ln-MEHHP in the AWHS cohort (N = 1124). Models adjusted for age, alcohol intake, physical activity, energy intake, smoking status, hypertension, dyslipidaemia, diabetes, work type, and work shift. The solid and long-dashed lines represent the estimated odds ratios and their 95% confidence intervals, respectively.

When the dose–response relationship was depicted using RCS, a positive but non-linear association was observed between ln-MEOHP and ln-MEHHP with general obesity (Figure 2).

## 4. Discussion

In this study conducted among Spanish adult males, we observed that urinary concentrations of MEOHP, MEHHP and ∑DEHP were associated with an increased prevalence of general obesity. Only MEHHP showed a monotonic trend, indicating a linear dose–response relationship on a logarithmic scale. In addition, MiBP was inversely associated with abdominal obesity. As expected, due to their high correlation as downstream metabolites of DEHP (Rho = 0.97), results for MEOHP and MEHHP were similar.

Our findings align with other key cross-sectional studies assessing the relationship between urinary phthalate metabolite concentrations and obesity among adults [22,24,25]. The positive association that we observed between MEOHP and MEHHP and general obesity is in keeping with NHANES (2007–2010) by Buser et al. [25] in which MEOHP and MEHHP were both associated with general obesity in males and female aged ≥ 20 years. Moreover, Li et al. [47] found a positive association between MEOHP and general obesity among Chinese males aged ≥ 60 years old, while in a comprehensive meta-analysis by Wu et al. [48], MEHHP showed a positive correlation with general obesity among adults. Furthermore, NHANES (1999–2002) by Hatch et al. [22] demonstrated a positive relationship between both MEOHP and MEHHP and BMI among 20- to 59-year-old American males. Our findings provide supporting evidence for this association.

Notwithstanding this, these findings were not corroborated by some studies. For example, the Shanghai Food Consumption Survey (SHFCS) (2012–2014) by Dong et al. [23] reported a positive association between both MEOHP and MEHHP and general obesity among women, but not among men. This result was replicated for MEHHP among males aged ≥ 19 years old in the Korean National Environmental Health Survey (KoNEHS) (2012–2014) by Kang et al. [31]. Another discrepancy between our findings and those of previous studies is that we did not find an association between MEOHP and MEHHP and abdominal obesity, while results from NHANES (1999–2002) by Stahlhut et al. [24] reported higher WC among males with greater urinary concentrations of these two metabolites. The conflicting results could be due to methodological differences; for example, some studies adjust for different covariates or apply creatinine correction to phthalate metabolites after principal analyses. Furthermore, variations between different age groups, sex, and race may increase heterogeneity [24,30,49]. This cumulative evidence highlights the important role of demographic differences and distinct study designs when assessing these exposures and associations.

DEHP is found in high quantities in plastic-packaged food products, especially fatty foods [50]. Foods containing a high content of fat (for example, meat and dairy) are more likely to absorb phthalates from packaging [20]. Our study found that those with higher urinary concentrations of ∑DEHP and its metabolites had a significantly higher calorie intake, suggesting that high-calorie food (especially ultra-processed food) could be the source of consumption. Even after adjustment for calorie intake and physical activity, the association with obesity was significant, indicating potentially obesogenic effects beyond energy balance.

The remaining two DEHP metabolites in our study, MECPP and MCMHP, showed positive associations with general obesity but failed to reach statistical significance (*p* = 0.062 and *p* = 0.551, respectively). Dong et al. [23] and Buser et al. [25] reported significant positive associations between MECPP and abdominal and general obesity, respectively, as shown in NHANES (2013–2014) by Zhang et al. [26] among Americans aged ≥ 20 years. In our dataset, these phthalate metabolites were highly correlated with ∑DEHP (Rho for MECPP = 0.9822; Rho for MCMHP = 0.9123) and maintained positive associations with general obesity. One possible explanation for this discrepancy is the variation in sample sizes between our study and that of NHANES. Regarding MCMHP, to our knowledge, its association with obesity in adults has not been assessed in previous research, except for its inclusion in the molar sum of DEHP in Dong et al. [23].

∑DEHP (weighted molar sum of urinary metabolites of DEHP) was positively associated with general obesity in this study. Results from NHANES (2007–2010) by Buser et al. [25] were similar, in that ∑DEHP was significantly associated with general obesity in older males (≥60 years). Furthermore, NHANES (2001–2010) by James-Todd et al. [51] also showed a positive relationship between ∑DEHP and abdominal obesity in 20- to 59-year-old American males. Previous studies have shown that DEHP exhibits anti-androgenic associations, and higher DEHP exposure in males has been linked to lower free testosterone levels [52], which in turn are associated with an increase in adiposity in males [53]. A further study found that, in humans, DEHP also significantly elevated leptin levels and interfered with fatty acid metabolism and lipid storage [54].

Contrary to our findings, NHANES (2013–2016) by Zhang et al. [55] showed that the sum of MEOHP, MEHHP, and MECPP was negatively associated with general obesity in the general US population. The variable used resembles ∑DEHP in our study (except lacking MCMHP). These mixed results should be interpreted with caution because of differences in phthalate metabolite concentration levels and the inclusion of both sexes in the NHANES study.

The inverse association observed between urinary MiBP levels and abdominal obesity may be influenced by specific occupational or behavioural factors rather than indicating a true protective effect. In our cohort of male car assembly plant workers, MiBP was the second most prevalent phthalate metabolite. It is a metabolite of diisobutyl phthalate (DiBP), a substance commonly found in industrial coatings, adhesives, and polymer-based materials. According to the European Chemicals Agency (ECHA) [56], DiBP can be released during industrial processes, particularly from materials with a high emission potential, such as tyres and brake pads. In this occupational context, therefore, workplace exposure could contribute to the overall phthalate burden. Differences in job type or the use of personal care products could influence exposure patterns. These variations may reflect underlying socioeconomic, lifestyle, or hygiene-related behaviours, which may also correlate with obesity prevalence. Additionally, like other endocrine-disrupting chemicals, MiBP may exhibit non-monotonic dose–response relationships, whereby different exposure levels produce different biological effects. Together, these factors may help to explain the inverse association observed, which warrants further investigation in future studies.

This study has several strengths. Firstly, because phthalate metabolites are produced in the human body, the samples collected could not have been contaminated by additional exposure to plastics. Second, the sample size was relatively large, with more than 1000 participants. Thirdly, the sample was well characterised for cardiovascular risk factors, including obesity, which was assessed using standardised methods. Finally, the study population is relatively homogeneous, consisting of male adults of similar age, origin and socio-economic background, which enhances the control of confounding factors.

This study also presents some limitations. Firstly, the cross-sectional design does not allow for establishing causal or temporal relationships. Secondly, the lipophilic properties of phthalates suggest that fat mass may be a more accurate physiological measure than WC and BMI. Thirdly, phthalates are rapidly metabolised by the human body, so it is possible that the urinary concentrations may only reflect short-term exposure. Finally, non-linear associations were found between some phthalate metabolites and obesity. A cautious interpretation is needed since they may reflect causal effects; however, we cannot rule out the existence of co-exposure.

## 5. Conclusions

In conclusion, this study provides supportive evidence for the potential obesogenic effects of DEHP and its metabolites (MEOHP and MEHHP) in adult males. These findings suggest that phthalates may contribute to the development of obesity and highlight the need for policy interventions to restrict the industrial use of these chemicals. Future research should focus on elucidating the mechanisms by which DEHP and its metabolites influence obesity beyond energy balance. Given that exposure to phthalates is largely unintentional and, due to their ubiquity, affects virtually all human populations, regulatory action to reduce unintentional exposure to these potentially harmful substances is both necessary and urgent.

## Figures and Tables

**Table 1:** Summary of participant characteristics (Aragon Workers’ Health Study, 2011–2014) N = 1124.

Characteristic	N = 1124		MEOHP			MEHHP			∑DEHP *	
		Q1	Q4	*p*-Value	Q1	Q4	*p*-Value	Q1	Q4	*p*-Value
**Urinary creatinine concentration (mg/dL)**	154.4 (57.1)	152.5 (62.4)	152.0 (54.6)	0.803	150.9 (60.7)	155.7 (55.9)	0.481	153.4 (62.4)	153.3 (54.9)	0.898
**Age (years), mean (SD)**	50.7 (3.66)	50.7 (3.63)	50.9 (3.74)	0.541	50.6 (3.68)	50.8 (3.69)	0.565	50.7 (3.68)	50.7 (3.75)	0.732
**Alcohol (g/day)**	22.5 (21.3)	21.1 (20.7)	24.0 (22.7)	0.081	19.9 (19.6)	24.5 (22.9)	**0.005**	21.0 (20.5)	24.0 (22.5)	**0.046**
**Physical activity (total METs/week)**	35.3 (21.5)	36.2 (21.5)	34.3 (21.0)	0.612	35.2 (21.4)	35.7 (21.5)	0.971	35.9 (21.8)	35.7 (22.0)	0.853
**Energy intake (kcal/day)**	2945.1 (764.9)	2850.1 (795.3)	3011.9 (754.2)	**0.022**	2862.8 (832.8)	2980.4 (724.9)	**0.046**	2855.1 (787.0)	2976.4 (736.1)	**0.045**
**Smoking status, *n* (%)**				0.071			**0.034**			0.115
No smoker	255 (22.7)	83 (32.6)	54 (21.2)		84 (32.9)	53 (20.8)		78 (30.6)	49 (19.2)	
Former smoker	487 (43.3)	110 (22.6)	123 (25.3)		105 (21.6)	129 (26.5)		108 (22.2)	130 (26.7)	
Current smoker	382 (34.0)	88 (23.0)	104 (27.2)		92 (24.1)	99 (25.9)		95 (24.9)	102 (26.7)	
**Hypertension**				0.870			0.662			0.913
No hypertension	682 (60.7)	170 (24.9)	173 (25.4)		172 (25.2)	176 (25.8)		170 (24.9)	173 (25.34)	
Hypertension	442 (39.3)	111 (25.1)	108 (24.4)		109 (24.7)	105 (23.8)		111 (25.1)	108 (24.4)	
**Dyslipidaemia**				**0.029**			0.145			0.130
No dyslipidaemia	390 (34.7)	86 (22.1)	110 (28.2)		94 (24.1)	107 (27.4)		87 (22.3)	108 (27.7)	
Dyslipidaemia	734 (65.3)	195 (26.6)	171 (23.3)		187 (25.5)	174 (23.7)		194 (26.4)	173 (23.6)	
**Diabetes**				0.635			0.722			0.906
No diabetes	1064 (94.7)	268 (25.2)	271 (25.5)		268 (25.2)	271 (25.5)		267 (25.1)	269 (25.3)	
Diabetes	60 (5.34)	13 (21.7)	10 (16.7)		13 (21.7)	10 (16.7)		14 (23.3)	12 (20.0)	
**Work type**				**0.007**			**0.018**			**0.001**
Sedentary work (office)	141 (12.5)	42 (29.8)	24 (17.0)		39 (27.7)	25 (17.7)		41 (29.1)	18 (12.8)	
Manual labour (assembly line)	983 (87.5)	239 (24.3)	257 (26.1)		242 (24.6)	256 (26.0)		240 (24.4)	263 (26.8)	
**Work shift**				**0.045**			**0.041**			**0.010**
Rotating: morning–afternoon	689 (61.3)	168 (24.4)	186 (27.0)		167 (24.2)	185 (26.9)		167 (24.2)	190 (27.6)	
Rotating: morning–afternoon–night	241 (21.4)	51 (21.2)	55 (22.8)		53 (22.0)	56 (23.2)		52 (21.6)	54 (22.4)	
Central	96 (8.54)	31 (32.3)	17 (17.7)		27 (28.1)	17 (17.7)		31 (32.3)	13 (13.5)	
Night	98 (8.72)	31 (31.6)	23 (23.5)		34 (34.7)	23 (23.5)		31 (31.6)	24 (24.5)	

Bolded *p*-values are statistically significant according to linear regression for continuous variables and logistic regression for categorical variables the exception of smoking status and work shift (Pearson’s chi-squared test). * ∑DEHP: sum of MEOHP, MEHHP, MECPP, and MCMHP.

**Table 2:** Correlations (Spearman) between urinary phthalate metabolites concentrations.

	∑DEHP *	MEP	MiBP	MnBP	MBzP	MEOHP	MECPP	MEHHP	MCMHP	OH-MiNP	cx-MiDP	OH-MiDP
**∑DEHP**	1.000											
**MEP**	0.128	1.000										
**MiBP**	0.010	0.054	1.000									
**MnBP**	0.004	0.010	0.048	1.000								
**MBzP**	0.115	0.090	0.115	0.023	1.000							
**MEOHP**	**0.979**	0.111	0.082	0.000	0.091	1.000						
**MECPP**	**0.982**	0.151	0.120	0.005	0.128	**0.933**	1.000					
**MEHHP**	**0.996**	0.118	0.096	0.004	0.114	**0.973**	**0.970**	1.000				
**MCMHP**	**0.912**	0.107	0.090	0.003	0.134	**0.878**	**0.893**	**0.899**	1.000			
**OH-MiNP**	0.081	0.051	0.047	−0.004	0.146	0.058	0.103	0.078	0.063	1.000		
**cx-MiDP**	0.036	0.009	0.027	−0.002	0.081	0.019	0.054	0.0300	0.046	0.219	1.000	
**OH-MiDP**	0.068	0.035	0.046	0.005	0.142	0.046	0.088	0.065	0.064	0.319	**0.826**	1.000

Bolded values signify Rho > 0.800. * ∑DEHP: molar sum of MEOHP, MEHHP, MECPP, and MCMHP.

**Table 3:** Odds ratios (95% CI) of urinary creatinine-corrected concentrations (μg/g creatinine) of phthalate metabolites and ΣDEHP in relation to abdominal obesity in the AWHS cohort (N = 1084).

Phthalate Metabolites as Continuous ^			Phthalate Metabolites in Quartiles				
Phthalate Metabolites (µg/g Creatinine)	OR (95% CI) for Every 1-ln Increase	*p*-Value	Q1 OR (95% CI)	Q2 OR (95% CI)	Q3 OR (95% CI)	Q4 OR (95% CI)	*p*-Test for Linear Trend
**MEP**							
	Events/*n*	317/1084		88/272	70/270	69/272	90/270
	Crude model	1.02 (0.92, 1.15)	0.678	1 (Ref.)	0.73 (0.50, 1.06)	0.71 (0.49, 1.03)	1.05 (0.73, 1.50)
	Adjusted model *	1.03 (0.91, 1.15)	0.682	1 (Ref.)	0.70 (0.47, 1.04)	0.69 (0.46, 1.03)	1.03 (0.70, 1.51)
**MiBP**							
	Events/*n*	317/1084		98/271	77/272	79/271	63/270
	Crude model	0.68 (0.54, 0.86)	**0.001**	1 (Ref.)	0.68 (0.49, 1.00)	0.73 (0.51, 1.04)	**0.54 (0.37, 0.78)**
	Adjusted model	0.73 (0.57, 0.93)	**0.010**	1 (Ref.)	0.73 (0.49, 1.07)	0.81 (0.55, 1.19)	**0.55 (0.37, 0.83)**
**MnBP**							
	Events/*n*	317/1084		82/269	86/70	72/273	77/272
	Crude model	0.89 (0.73, 1.09)	0.269	1 (Ref.)	1.07 (0.74, 1.53)	0.82 (0.56, 1.19)	0.90 (0.62, 1.30)
	Adjusted model	0.92 (0.74, 1.14)	0.442	1 (Ref.)	1.17 (0.79, 1.73)	0.84 (0.56, 1.25)	0.95 (0.64, 1.42)
**MBzP**							
	Events/*n*	317/1084		73/272	93/271	81/273	70/268
	Crude model	0.99 (0.84, 1.18)	0.953	1 (Ref.)	1.42 (0.99, 2.06)	1.15 (0.79, 1.67)	0.96 (0.66, 1.41)
	Adjusted model	1.02 (0.85, 1.22)	0.861	1 (Ref.)	**1.53 (1.04, 2.27)**	1.26 (0.84, 1.87)	0.99 (0.66, 1.49)
**MEOHP**							
	Events/*n*	317/1084		65/273	99/268	72/271	81/272
	Crude model	1.09 (0.91, 1.31)	0.342	1 (Ref.)	**1.87 (1.29, 2.72)**	1.16 (0.79, 1.71)	1.36 (0.93, 1.99)
	Adjusted model	1.17 (0.96, 1.42)	0.119	1 (Ref.)	**1.99 (1.33, 2.96)**	1.24 (0.82, 1.88)	**1.53 (1.02, 2.30)**
**MECPP**							
	Events/*n*	317/1084		73/274	88/270	72/268	84/272
	Crude model	1.11 (0.92, 1.34)	0.274	1 (Ref.)	1.33 (0.92, 1.93)	1.01 (0.69, 1.48)	1.23 (0.85, 1.78)
	Adjusted model	1.14 (0.93, 1.40)	0.204	1 (Ref.)	1.27 (0.86, 1.88)	0.97 (0.65, 1.46)	1.28 (0.86, 1.90)
**MEHHP**							
	Events/*n*	317/1084		70/273	87/270	78/269	82/272
	Crude model	1.10 (0.91, 1.31)	0.324	1 (Ref.)	1.38 (0.95, 2.00)	1.18 (0.81, 1.73)	1.25 (0.86, 1.82)
	Adjusted model	1.18 (0.97, 1.43)	0.096	1 (Ref.)	1.42 (0.95, 2.11)	1.24 (0.82, 1.86)	1.43 (0.96, 2.13)
**MCMHP**							
	Events/*n*	317/1084		77/272	91/268	66/273	83/271
	Crude model	1.01 (0.82, 1.24)	0.950	1 (Ref.)	1.30 (0.90, 1.88)	0.81 (0.55, 1.18)	1.12 (0.77, 1.62)
	Adjusted model	1.05 (0.84, 1.31)	0.686	1 (Ref.)	1.44 (0.97, 2.14)	0.86 (0.57, 1.29)	1.19 (0.80, 1.77)
**∑DEHP ^†^**							
	Events/*n*	317/1084		72/274	88/269	73/268	84/273
	Crude model	1.10 (0.91, 1.34)	0.320	1 (Ref.)	1.36 (0.94, 1.98)	1.05 (0.72, 1.54)	1.25 (0.86, 1.81)
	Adjusted model	1.16 (0.95, 1.43)	0.152	1 (Ref.)	1.46 (0.98, 2.16)	1.04 (0.69, 1.56)	1.40 (0.94, 2.09)
**cx-MiDP**							
	Events/*n*	317/1084		78/273	74/272	87/270	78/269
	Crude model	1.04 (0.87, 1.25)	0.642	1 (Ref.)	0.93 (0.64, 1.36)	1.19 (0.82, 1.71)	1.02 (0.70, 1.48)
	Adjusted model	1.06 (0.87, 1.29)	0.578	1 (Ref.)	0.94 (0.63, 1.41)	1.17 (0.79, 1.73)	1.00 (0.67, 1.49)
**OH-MiDP**							
	Events/*n*	317/1084		73/272	83/271	80/269	81/272
	Crude model	1.03 (0.87, 1.22)	0.722	1 (Ref.)	1.20 (0.83, 1.75)	1.15 (0.79, 1.68)	1.16 (0.80, 1.68)
	Adjusted model	1.12 (0.93, 1.35)	0.224	1 (Ref.)	1.31 (0.88, 1.95)	1.19 (0.79, 1.77)	1.36 (0.91, 2.04)
**OH-MiNP**							
	Events/*n*	317/1084		82/273	67/269	83/269	85/273
	Crude model	1.08 (0.94, 1.24)	0.280	1 (Ref.)	0.77 (0.53, 1.13)	1.04 (0.72, 1.50)	1.05 (0.73, 1.52)
	Adjusted model	1.10 (0.95, 1.28)	0.205	1 (Ref.)	0.80 (0.53, 1.20)	1.06 (0.71, 1.57)	1.12 (0.76, 1.65)

OR: odds ratio; CI: confidence interval. Bolded *p*-values < 0.05. * Adjusted for age, alcohol intake, physical activity, energy intake, smoking status, hypertension, dyslipidaemia, diabetes, work type and work shift. ^ log-transformed phthalate metabolites. ^†^ ∑DEHP: molar sum of MEOHP, MEHHP, MECPP, and MCMHP.

**Table 4:** Odds ratios (95% CI) of urinary creatinine-corrected concentrations (μg/g creatinine) of phthalate metabolites and ΣDEHP in relation to general obesity in the AWHS cohort (N = 1124).

Phthalate Metabolites as Continuous ^			Phthalate Metabolites in Quartiles				
Phthalate Metabolites (µg/g-Creatinine)	OR (95% CI) for Every 1-ln Increase	*p*-Value	Q1 OR (95% CI)	Q2 OR (95% CI)	Q3 OR (95% CI)	Q4 OR (95% CI)	*p*-Test for Linear Trend
**MEP**							
	Events/*n*	246/1124		65/281	57/281	54/281	70/281
	Crude model	1.06 (0.94, 1.19)	0.376	1 (Ref.)	0.85 (0.57, 1.26)	0.79 (0.53, 1.19)	1.10 (0.75, 1.62)
	Adjusted model *	1.07 (0.95, 1.22)	0.272	1 (Ref.)	0.86 (0.56, 1.31)	0.78 (0.51, 1.21)	1.13 (0.74, 1.70)
**MiBP**							
	Events/*n*	246/1124		73/281	62/281	59/281	52/281
	Crude model	0.85 (0.67, 1.08)	0.178	1 (Ref.)	0.81 (0.55, 1.19)	0.76 (0.51, 1.12)	**0.65 (0.43, 0.97)**
	Adjusted model	0.91 (0.71, 1.17)	0.485	1 (Ref.)	0.87 (0.57, 1.31)	0.85 (0.56, 1.29)	0.68 (0.44, 1.04)
**MnBP**							
	Events/*n*	246/1124		64/281	60/281	57/281	65/281
	Crude model	0.97 (0.79, 1.19)	0.765	1 (Ref.)	0.92 (0.62, 1.37)	0.86 (0.58, 1.29)	1.02 (0.69, 1.51)
	Adjusted model	0.97 (0.78, 1.21)	0.788	1 (Ref.)	0.95 (0.62, 1.45)	0.87 (0.57, 1.34)	1.03 (0.68, 1.58)
**MBzP**							
	Events/*n*	246/1124		62/281	61/281	58/281	65/281
	Crude model	1.06 (0.88, 1.26)	0.557	1 (Ref.)	0.98 (0.66, 1.46)	0.92 (0.61, 1.38)	1.06 (0.72, 1.58)
	Adjusted model	1.06 (0.88, 1.29)	0.523	1 (Ref.)	0.96 (0.63, 1.47)	0.95 (0.62, 1.46)	1.03 (0.68, 1.58)
**MEOHP**							
	Events/*n*	246/1124		48/281	73/281	58/281	67/281
	Crude model	1.17 (0.96, 1.43)	0.110	1 (Ref.)	**1.70 (1.13, 2.57)**	1.26 (0.83, 1.93)	**1.52 (1.00, 2.30)**
	Adjusted model	1.24 (1.00, 1.53)	**0.047**	1 (Ref.)	**1.79 (1.15, 2.76)**	1.29 (0.82, 2.03)	**1.70 (1.09, 2.64)**
**MECPP**							
	Events/*n*	246/1124		53/281	64/281	57/281	72/281
	Crude model	1.21 (0.99, 1.48)	0.066	1 (Ref.)	1.27 (0.84, 1.91)	1.09 (0.72, 1.66)	1.48 (0.99, 2.21)
	Adjusted model	1.23 (0.99, 1.53)	0.062	1 (Ref.)	1.20 (0.78, 1.86)	1.03 (0.66, 1.60)	1.51 (0.98, 2.32)
**MEHHP**							
	Events/*n*	246/1124		51/281	64/281	62/281	69/281
	Crude model	1.19 (0.98, 1.45)	0.075	1 (Ref.)	1.33 (0.88, 2.01)	1.28 (0.84, 1.93)	1.47 (0.98. 2.21)
	Adjusted model	1.26 (1.03, 1.55)	**0.027**	1 (Ref.)	1.32 (0.86, 2.06)	1.28 (0.82, 1.99)	**1.63 (1.06, 2.52)**
**MCMHP**							
	Events/*n*	246/1124		64/281	62/281	52/281	68/281
	Crude model	1.01 (0.80, 1.26)	0.961	1 (Ref.)	0.96 (0.65, 1.43)	0.77 (0.51, 1.16)	1.08 (0.73, 1.60)
	Adjusted model	1.08 (0.85, 1.37)	0.551	1 (Ref.)	1.07 (0.70, 1.64)	0.84 (0.54, 1.30)	1.20 (0.79, 1.83)
**∑DEHP ^†^**							
	Events/*n*	246/1124		52/281	62/281	64/281	68/281
	Crude model	1.21 (0.98, 1.48)	0.071	1 (Ref.)	1.25 (0.83, 1.88)	1.30 (0.86, 1.96)	1.41 (0.94, 2.11)
	Adjusted model	1.26 (1.01, 1.58)	**0.038**	1 (Ref.)	1.31 (0.85, 2.03)	1.27 (0.82, 1.96)	1.54 (0.99, 2.37)
**cx-MiDP**							
	Events/*n*	246/1124		70/281	56/281	63/281	57/281
	Crude model	0.90 (0.73, 1.10)	0.287	1 (Ref.)	0.75 (0.50, 1.12)	0.87 (0.59, 1.29)	0.77 (0.52, 1.14)
	Adjusted model	0.87 (0.70, 1.09)	0.233	1 (Ref.)	0.76 (0.50, 1.17)	0.82 (0.54, 1.25)	0.70 (0.46, 1.08)
**OH-MiDP**							
	Events/*n*	246/1124		59/281	59/281	64/281	64/281
	Crude model	1.01 (0.84, 1.22)	0.892	1 (Ref.)	1.00 (0.67, 1.50)	1.11 (0.74, 1.66)	1.11 (0.74, 1.66)
	Adjusted model	1.05 (0.86, 1.28)	0.649	1 (Ref.)	1.03 (0.67, 1.59)	1.11 (0.72, 1.69)	1.15 (0.75, 1.77)
**ohminp**							
	Events/*n*	246/1124		63/281	53/281	65/281	65/281
	Crude model	1.10 (0.94, 1.28)	0.227	1 (Ref.)	0.80 (0.53, 1.21)	1.04 (0.70, 1.54)	1.04 (0.70, 1.54)
	Adjusted model	1.10 (0.94, 1.29)	0.251	1 (Ref.)	0.81 (0.53, 1.25)	1.02 (0.67, 1.56)	1.04 (0.68, 1.58)

OR: odds ratio; CI: confidence interval. Bolded *p*-values < 0.05. * Adjusted for age, alcohol intake, physical activity, energy intake, smoking status, hypertension, dyslipidaemia, diabetes, work type, and work shift. ^ log-transformed phthalate metabolites. ^†^ ∑DEHP: molar sum of MEOHP, MEHHP, MECPP, and MCMHP.

## Data Availability

Data are available for research purposes upon specific request.

## Supplementary Materials

The following supporting information can be downloaded at: https://www.mdpi.com/article/10.3390/nu17111869/s1, Table S1. Sensitivity analysis of the association between phthalate metabolites and abdominal obesity. Table S2. Sensitivity analysis of the association between phthalate metabolites and general obesity.

## Abbreviations

The following abbreviations are used in this manuscript:

AWHS	Aragon Workers’ Health Study
BMI	Body mass index
cx-MiDP	Mono-carboxy isononyl phthalate
DEHP	Di(2-ethylhexyl) phthalate
DIBP	Diisobutyl phthalate
MBzP	Monobenzyl phthalate
MCMHP	Mono(2-carboxymethylhexyl) phthalate
MECPP	Mono-(2-ethyl-5-carboxypentyl) phthalate
MEHHP	Mono-(2-ethyl-5-hydroxyhexyl) phthalate
MEOHP	Mono-(2-ethyl-5-oxohexyl) phthalate
MEP	Monoethyl phthalate
MiBP	Mono-isobutyl phthalate
MnBP	Mono-n-butyl phthalate
NHANES	National Health and Nutrition Examination Survey
OH-MiDP	Mono-hydroxy-isodecyl phthalate
OH-MiNP	Mono-hydroxy-isononyl phthalate
WC	Waist circumference
