# Supplementary material for: Phthalate Metabolites and Their Relationship with Abdominal and General Obesity: Evidence from the Aragon Workers’ Health Study (AWHS)
Source: Nutrients. 2025 May 30;17(11):1869. doi: 10.3390/nu17111869 (PMC12157022; doi:10.3390/nu17111869)
Supplement: Supplementary file 1 [file nutrients-17-01869-s001.zip › nutrients-3656084-supplementary.pdf]

Article

Phthalate Metabolites and Their Relationship with Abdominal and General Obesity: Evidence from the Aragon Workers' Health Study (AWHS)

Jordan Akritidis [1], Diana María Mérida [1,2,3], Carolina Torrijo-Belanche [4], Belén Moreno-Franco [4,5,6], Sofía Gimeno-Ruiz [7], Jimena Rey-García [8], María Morales-Suarez-Varela [2,9] and Pilar Guallar-Castillón [1,2,10,*]

[1] Department of Preventive Medicine and Public Health, School of Medicine, Universidad Autónoma de Madrid, Arzobispo Morcillo 4, 28029 Madrid, Spain; jordan.akritidis@outlook.com (J.A.); dianamerida26@gmail.com (D.M.M.)
[2] CIBERESP (CIBER of Epidemiology and Public Health), 28029 Madrid, Spain; maria.m.morales@uv.es
[3] Fundación Teófilo Hernando, Las Rozas de Madrid, 28290 Madrid, Spain
[4] Department of Preventive Medicine and Public Health, Universidad de Zaragoza, 50009 Zaragoza, Spain; carolinatorrijob@gmail.com (C.T.-B.); belenmorenofranco@gmail.com (B.M.-F.)
[5] Instituto de Investigación Sanitaria Aragón, Hospital Universitario Miguel Servet, 50009 Zaragoza, Spain
[6] CIBERCV (CIBER de Enfermedades Cardiovasculares), 28029 Madrid, Spain
[7] Veterinary School, Universidad de Zaragoza, 50013 Zaragoza, Spain; gimenoruizsofia@gmail.com
[8] Department of Internal Medicine, Hospital Universitario Rey Juan Carlos, Instituto de Investigación Sanitaria-Fundación Jiménez Díaz (IIS-FJD), 28933 Móstoles, Spain; jimena.reygarcia@gmail.com
[9] Departament de Medicina Preventiva i Salut Pública, Ciències d'Alimentació, Toxicologia i Medicina Legal, Universitat de València, 46003 Valencia, Spain
[10] Instituto Madrileño de Estudios Avanzados en Alimentación (IMDEA)-Food Institute, Campus de Excelencia Internacional Universidad Autónoma de Madrid + Consejo Superior de Investigaciones Científicas (CEI UAM + CSIC), Carretera de Cantoblanco 8, 28049 Madrid, Spain
* Correspondence: mpilar.guallar@uam.es

Supplementary Data

Table S1. Sensitivity analysis of the association between phthalate metabolites and abdominal obesity.

Table S2. Sensitivity analysis of the association between phthalate metabolites and general obesity.

Table S1. Odds ratios (95% CI) of concentrations (ng/mL) of phthalate metabolites and ΣDEHP in relation to abdominal obesity in the AWHS cohort (N=1,084).

	Phthalate metabolites as continuous^		Phthalate metabolites in quartiles				
**Phthalate metabolites (µg/g creatinine)**	**OR (95% CI) for every 1-ln increase**	***p*-value**	**Q1 OR (95% CI)**	**Q2 OR (95% CI)**	**Q3 OR (95% CI)**	**Q4 OR (95% CI)**	***p*-test for linear trend**
**MEP**							
events/n	317/1084		88/272	70/270	69/272	90/270	
Crude model*	1.02 (.091, 1.14)	0.712	1 (Ref.)	0.79 (0.55, 1.15)	0.70 (0.48, 1.03)	1.04 (0.71, 1.51)	0.985
Adjusted model	1.02 (0.91, 1.15)	0.742	1 (Ref.)	0.79 (0.53, 1.18)	0.70 (0.46, 1.05)	1.04 (0.69, 1.55)	0.997
**MiBP**							
events/n	317/1084		98/271	77/272	79/271	63/270	
Crude model	0.68 (0.54, 0.85)	**0.001**	1 (Ref.)	1.01 (0.70, 1.48)	0.73 (0.48, 1.11)	**0.51 (0.32, 0.82)**	**0.002**
Adjusted model	0.71 (0.56, 0.91)	**0.006**	1 (Ref.)	0.98 (0.65, 1.47)	0.79 (0.51, 1.23)	**0.52 (0.32, 0.86)**	**0.007**
**MnBP**							
events/n	317/1084		82/269	86/70	72/273	77/272	
Crude model	0.88 (0.72, 1.08)	0.234	1 (Ref.)	1.06 (0.73, 1.55)	0.68 (0.45, 1.03)	0.88 (0.57, 1.35)	0.233
Adjusted model	0.91 (0.73, 1.13)	0.382	1 (Ref.)	1.16 (0.78, 1.74)	0.69 (0.44, 1.07)	0.94 (0.59, 1.49)	0.321
**MBzP**							
events/n	317/1084		73/272	93/271	81/273	70/268	
Crude model	0.99 (0.84, 1.17)	0.898	1 (Ref.)	1.17 (0.81, 1.70)	1.02 (0.69, 1.50)	0.84 (0.55, 1.27)	0.296
Adjusted model	1.01 (0.85, 1.21)	0.904	1 (Ref.)	1.18 (0.79, 1.76)	1.10 (0.72, 1.67)	0.86 (0.55, 1.35)	0.462
**MEOHP**							
events/n	317/1084		65/273	99/268	72/271	81/272	
Crude model	1.08 (0.90, 1.30)	0.390	1 (Ref.)	1.12 (0.76, 1.66)	1.10 (0.73, 1.66)	1.12 (0.74, 1.71)	0.646
Adjusted model	1.15 (0.95, 1.40)	0.144	1 (Ref.)	1.13 (0.74, 1.72)	1.25 (0.81, 1.94)	1.21 (0.76, 1.90)	0.372
**MECPP**							
events/n	317/1084		73/274	88/270	72/268	84/272	
Crude model	1.10 (0.91, 1.33)	0.309	1 (Ref.)	1.04 (0.70, 1.54)	0.93 (0.61, 1.41)	1.26 (0.83, 1.91)	0.327
Adjusted model	1.13 (0.93, 1.39)	0.223	1 (Ref.)	1.03 (0.68, 1.58)	0.91 (0.58, 1.42)	1.28 (0.82, 2.00)	0.340
**MEHHP**							
events/n	317/1084		70/273	87/270	78/269	82/272	
Crude model	1.08 (0.91, 1.29)	0.376	1 (Ref.)	1.08 (0.73, 1.60)	0.94 (0.62, 1.42)	1.18 (0.78, 1.80)	0.567
Adjusted model	1.16 (0.96, 1.40)	0.127	1 (Ref.)	1.14 (0.75, 1.74)	1.05 (0.67, 1.65)	1.33 (0.84, 2.09)	0.280
**MCMHP**							
events/n	317/1084		77/272	91/268	66/273	83/271	
Crude model	1.00 (0.81, 1.23)	0.989	1 (Ref.)	0.86 (0.58, 1.27)	0.90 (0.60, 1.36)	0.96 (0.64, 1.46)	0.980
Adjusted model	1.05 (0.84, 1.30)	0.694	1 (Ref.)	0.95 (0.62, 1.44)	0.95 (0.61, 1.48)	1.04 (0.66, 1.63)	0.836
**∑DEHP†**							
events/n	317/1084		72/274	88/269	73/268	84/273	
Crude model	1.09 (0.90, 1.32)	0.366	1 (Ref.)	1.22 (0.82, 1.81)	0.89 (0.58, 1.35)	1.27 (0.83, 1.93)	0.562
Adjusted model	1.15 (0.94, 1.41)	0.178	1 (Ref.)	1.22 (0.80, 1.87)	0.95 (0.60, 1.50)	1.34 (0.85, 2.11)	0.375

**cx-MiDP**							
events/n	317/1084		78/273	74/272	87/270	78/269	
Crude model	1.05 (0.88, 1.27)	0.572	1 (Ref.)	0.93 (0.63, 1.36)	0.97 (0.66, 1.44)	1.08 (0.73, 1.60)	0.641
Adjusted model	1.10 (0.90, 1.35)	0.343	1 (Ref.)	0.92 (0.61, 1.38)	1.03 (0.67, 1.56)	1.11 (0.73, 1.70)	0.500
**OH-MiDP**							
events/n	317/1084		73/272	83/271	80/269	81/272	
Crude model	1.03 (0.87, 1.22)	0.718	1 (Ref.)	1.10 (0.75, 1.61)	0.79 (0.53, 1.18)	1.17 (0.79, 1.75)	0.775
Adjusted model	1.14 (0.95, 1.37)	0.167	1 (Ref.)	1.14 (0.76, 1.71)	0.84 (0.55, 1.29)	1.46 (0.55, 1.29)	0.222
**ohminp**							
events/n	317/1084		82/273	67/269	83/269	85/273	
Crude model	1.08 (0.94, 1.25)	0.274	1 (Ref.)	0.74 (0.51, 1.09)	0.76 (0.52, 1.13)	1.10 (0.75, 1.62)	0.492
Adjusted model	1.11 (0.96, 1.30)	0.157	1 (Ref.)	0.82 (0.54, 1.24)	0.82 (0.54, 1.25)	1.20 (0.80, 1.81)	0.334

OR: odds ratio; CI: confidence interval. Bolded *p*-values <0.05.

* Crude models were adjusted for creatinine only. Adjusted model were adjusted for creatinine, age, alcohol intake, physical activity, energy intake, smoking status, hypertension, dyslipidaemia, diabetes, work type and work shift.

^ log-transformed phthalate metabolites

† ∑DEHP: molar sum of MEOHP, MEHHP, MECPP and MCMHP.

Table S2. Odds ratios (95% CI) of concentrations (ng/mL) of phthalate metabolites and ΣDEHP in relation to general obesity in the AWHS cohort (N=1,124).

Phthalate metabolites (μg/g-creatinine)	Phthalate metabolites as continuous^ OR (95% CI) for every 1-ln increase	*p*-value	Phthalate metabolites in quartiles Q1 OR (95% CI)	Q2 OR (95% CI)	Q3 OR (95% CI)	Q4 OR (95% CI)	*p*-test for linear trend
**MEP**							
events/n	246/1124		65/281	57/281	54/281	70/281	
Crude model*	1.06 (0.94, 1.19)	0.376	1 (Ref.)	0.73 (0.48, 1.09)	**0.66 (0.43, 1.00)**	1.03 (0.69, 1.53)	0.950
Adjusted model	1.07 (0.94, 1.22)	0.300	1 (Ref.)	0.74 (0.48, 1.14)	0.66 (0.42, 1.02)	1.06 (0.69, 1.63)	0.890
**MiBP**							
events/n	246/1124		73/281	62/281	59/281	52/281	
Crude model	0.84 (0.67, 1.07)	0.165	1 (Ref.)	0.93 (0.62, 1.39)	0.56 (0.35, 0.88)	0.56 (0.34, 0.91)	0.005
Adjusted model	0.90 (0.70, 1.16)	0.409	1 (Ref.)	0.86 (0.56, 1.33)	0.57 (0.35, 0.93)	0.56 (0.33, 0.95)	0.013
**MnBP**							
events/n	246/1124		64/281	60/281	57/281	65/281	
Crude model	0.97 (0.79, 1.19)	0.764	1 (Ref.)	1.02 (0.67, 1.54)	0.84 (0.54, 1.31)	1.00 (0.63, 1.58)	0.793
Adjusted model	0.97 (0.78, 1.20)	0.762	1 (Ref.)	1.10 (0.71, 1.71)	0.88 (0.55, 1.42)	1.00 (0.60, 1.64)	0.765
**MBzP**							
events/n	246/1124		62/281	61/281	58/281	65/281	
Crude model	1.05 (0.88, 1.26)	0.558	1 (Ref.)	0.86 (0.57, 1.29)	0.85 (0.56, 1.29)	0.87 (0.56, 1.34)	0.559
Adjusted model	1.06 (0.88, 1.29)	0.523	1 (Ref.)	0.81 (0.53, 1.25)	0.86 (0.55, 1.35)	0.87 (0.55, 1.38)	0.646
**MEOHP**							
events/n	246/1124		48/281	73/281	58/281	67/281	
Crude model	1.17 (0.96, 1.42)	0.114	1 (Ref.)	1.45 (0.95, 2.24)	1.10 (0.70, 1.75)	**1.59 (1.01, 2.50)**	0.140
Adjusted model	1.23 (1.00, 1.51)	0.051	1 (Ref.)	1.48 (0.94, 2.33)	1.16 (0.71, 1.90)	**1.70 (1.05, 2.77)**	0.086
**MECPP**							
events/n	246/1124		53/281	64/281	57/281	72/281	
Crude model	1.21 (0.99, 1.47)	0.066	1 (Ref.)	1.31 (0.84, 2.02)	1.14 (0.72, 1.81)	**1.63 (1.04, 2.57)**	0.064
Adjusted model	1.23 (0.99, 1.53)	0.060	1 (Ref.)	1.31 (0.82, 2.09)	1.03 (0.63, 1.69)	**1.63 (1.00, 2.66)**	0.101
**MEHHP**							
events/n	246/1124		51/281	64/281	62/281	69/281	
Crude model	1.18 (0.98, 1.43)	0.082	1 (Ref.)	1.28 (0.83, 1.98)	1.03 (0.65, 1.64)	**1.60 (1.01, 2.53)**	0.091
Adjusted model	1.25 (1.02, 1.53)	**0.034**	1 (Ref.)	1.32 (0.83, 2.09)	1.06 (0.65, 1.75)	**1.77 (1.08, 2.88)**	**0.048**
**MCMHP**							
events/n	246/1124		64/281	62/281	52/281	68/281	
Crude model	1.01 (0.81, 1.26)	0.912	1 (Ref.)	0.61 (0.40, 0.95)	0.79 (0.51, 1.23)	0.87 (0.56, 1.37)	0.983
Adjusted model	1.08 (0.86, 1.37)	0.503	1 (Ref.)	0.67 (0.42, 1.06)	0.78 (0.49, 1.26)	0.99 (0.61, 1.60)	0.751
**∑DEHP†**							
events/n	246/1124		52/281	62/281	64/281	68/281	
Crude model	1.20 (0.98, 1.47)	0.074	1 (Ref.)	1.25 (0.81, 1.93)	0.88 (0.55, 1.40)	1.49 (0.94, 2.35)	0.213
Adjusted model	1.26 (1.01, 1.57)	**0.041**	1 (Ref.)	1.22 (0.77, 1.94)	0.85 (0.52, 1.41)	1.56 (0.95, 2.54)	0.167
**cx-MiDP**							

events/n	246/1124		70/281	56/281	63/281	57/281	
Crude model	0.92 (0.75, 1.12)	0.397	1 (Ref.)	0.84 (0.56, 1.27)	0.82 (0.53, 1.24)	0.83 (0.54, 1.27)	0.405
Adjusted model	0.92 (0.73, 1.15)	0.466	1 (Ref.)	0.82 (0.53, 1.27)	0.81 (0.51, 1.27)	0.78 (0.50, 1.24)	0.331
**OH-MiDP**							
events/n	246/1124		59/281	59/281	64/281	64/281	
Crude model	1.02 (0.85, 1.23)	0.804	1 (Ref.)	0.85 (0.56, 1.29)	0.75 (0.49, 1.15)	1.09 (0.71, 1.66)	0.766
Adjusted model	1.07 (0.88, 1.31)	0.477	1 (Ref.)	0.83 (0.53, 1.30)	0.73 (0.46, 1.16)	1.17 (0.74, 1.86)	0.572
**ohminp**							
events/n	246/1124		63/281	53/281	65/281	65/281	
Crude model	1.11 (0.95, 1.29)	0.189	1 (Ref.)	0.75 (0.49, 1.15)	0.94 (0.61, 1.43)	1.12 (0.74, 1.69)	0.360
Adjusted model	1.12 (0.95, 1.32)	0.174	1 (Ref.)	0.77 (0.49, 1.22)	0.94 (0.60, 1.48)	1.14 (0.73, 1.77)	0.369

OR: odds ratio; CI: confidence interval. Bolded *p*-values <0.05.

* Crude models were adjusted for creatinine only. Adjusted model were adjusted for creatinine, age, alcohol intake, physical activity, energy intake, smoking status, hypertension, dyslipidaemia, diabetes, work type and work shift.

^ log-transformed phthalate metabolites

† $\sum$DEHP: molar sum of MEOHP, MEHHP, MECPP and MCMHP.
